# Evaluation of Serum Biomarkers for Improved Diagnosis of Candidemia

**DOI:** 10.3390/jof11030224

**Published:** 2025-03-15

**Authors:** Amélie Kinet-Poleur, Corentin Deckers, Imane Saad Albichr, Pierre Bogaerts, Patrick M. Honoré, Pierre Bulpa, Nathalie Ausselet, Frederic Foret, François Kidd, Te-Din Huang, Isabel Montesinos

**Affiliations:** 1 Laboratory Medicine—Microbiology, CHU UCL Namur, 5530 Yvoir, Belgium; corentin.deckers@uclouvain.be (C.D.); imane.saadalbichr@chuuclnamur.uclouvain.be (I.S.A.); pierre.bogaerts@chuuclnamur.uclouvain.be (P.B.); te-din.huang@chuuclnamur.uclouvain.be (T.-D.H.); 2 Intensive Care Unit, CHU UCL Namur, 5530 Yvoir, Belgium; patrick.honore@chuuclnamur.uclouvain.be (P.M.H.); pierre.bulpa@chuuclnamur.uclouvain.be (P.B.); frederic.foret@chuuclnamur.uclouvain.be (F.F.); 3 Infectiology Department, CHU UCL Namur, 5530 Yvoir, Belgium; nathalie.ausselet@chuuclnamur.uclouvain.be (N.A.); francois.kidd@chuuclnamur.uclouvain.be (F.K.)

**Keywords:** candidemia, Wako β-D-glucan, CandId PCR, CAGTA VirClia, serum biomarkers

## Abstract

Background: Candidemia, associated with high morbidity and mortality, remains challenging to diagnose due to the limitations of blood cultures. Serological biomarkers offer faster detection, enabling earlier treatment and improving outcomes. Methods: This study, conducted at CHU UCL Namur (August 2023–January 2025), evaluated three diagnostic biomarkers for candidemia: CAGTA IgG VirClia Monotest, Wako β-D-glucan test, and CandId OLM RT-PCR. A total of 35 candidemia cases and 20 controls were included. Results: CAGTA IgG VirClia Monotest demonstrated low sensitivity (46%) and moderate specificity (75%). Both Wako β-D-glucan test and CandId OLM RT-PCR exhibited higher sensitivity (74% and 71%, respectively) and excellent specificity (100%). The combined use of Wako β-D-glucan test and CandId OLM RT-PCR further enhanced diagnostic performance, achieving 91% sensitivity and 100% specificity. Misidentification between *Candida albicans* and *Candida dubliniensis* was observed with CandId PCR, underlining a potential limitation in species-level discrimination. Conclusions: Both Wako β-D-glucan test and CandID OLM RT-PCR outperformed CAGTA IgG VirClia Monotest in diagnosing candidemia, and their combination significantly improved diagnostic accuracy, albeit at a higher cost. These findings highlight the potential of integrating multiple biomarkers into diagnostic workflows to optimize early detection, though further studies are needed to refine strategies and address challenges.

## 1. Introduction

The rising incidence of invasive fungal diseases is largely driven by an aging, immunocompromised population with multiple comorbidities. Candidemia, the most severe form of *Candida* infection, is associated with a mortality rate of nearly 50% in critical care settings and ranks as the third leading cause of sepsis [1,2]. Effective management relies on the timely initiation of antifungal therapy, as candidemia prolongs hospital stays and increases healthcare costs. Rapid and accurate diagnostics are essential to avoid delays in treatment [3,4].

Diagnosing candidemia is challenging due to the lack of specific symptoms, and blood cultures (BCs), while considered the gold standard, have limited sensitivity (~50%) and lengthy turnaround times. This often leads to empirical antifungal use, raising the risk of antifungal resistance. Furthermore, diagnostic accuracy varies across patient populations [5,6,7]. Current diagnostic tests for invasive candidiasis include BCs, direct examination, and non-culture-based assays such as mannan antigen and anti-*Candida* IgG/IgM tests. CAGTA detects IgG antibodies against the *Candida* mycelium cell wall and has been automated for use on the VirClia system. The (1-3)-β-D-glucan (BDG) assay, a near pan-fungal marker, is useful for ruling out candidiasis. Additionally, PCR assays in serum or plasma offer high sensitivity and specificity, detecting *Candida* DNA even when BCs are negative. However, PCR’s cost and technical requirements limit its accessibility in many labs [8,9,10].

The aim of this study is to evaluate the performances of three serum biomarkers for the diagnosis of candidemia: Invasive Candidiasis (CAGTA) IgG VirClia Monotest, Wako β-D-Glucan Test, and OLM CandId Real-Time PCR.

## 2. Materials and Methods

### 2.1. Institutional Setting and Patient Cohort

This research was conducted at the Medical Laboratory Department of CHU UCL Namur, a university hospital with 936 beds distributed across three sites: Dinant, Godinne, and Sainte-Elisabeth. From August 2023 to January 2025, we included 35 patients with positive BCs for *Candida* species and 20 patients with negative BCs within the same period. BCs were incubated in the BACT/ALERT^®^ VIRTUO^®^ (BioMérieux, Marcy l’Etoile, France) and the identification of the yeasts was carried out by matrix-assisted laser desorption/ionization time-of-flight mass spectrometry (Bruker BioSpin Corporation, San José, CA, USA). We reviewed medical records to extract demographic data, hospitalization unit, antifungal exposure at the time of sampling, and clinical evidence suggestive of deep-seated candidemia, along with other relevant clinical data required for the study. This study was approved by the institutional ethics committee, ensuring adherence to the highest ethical standards (CE Mont-Godinne 201/2023).

### 2.2. Invasive Candidiasis (CAGTA) IgG VirClia Monotest

The Invasive Candidiasis (CAGTA) IgG VirClia Monotest (Vircell Microbiologists, Granada, Spain) (CAGTA) detects the presence of anti-*Candida albicans* germ tube antibodies in serum samples. This assay employs automated chemiluminescence technology for the qualitative and quantitative detection of CAGTA IgG. Serum samples were collected from patients on the same day as the samples for BCs and stored at −20 °C until analysis. Before testing, samples were thawed at room temperature and thoroughly mixed. The assay was performed on the VirClia Lotus random-access analyzer, following the manufacturer’s instructions. Each test was run using a monotest strip format containing all necessary reagents, including controls, in a ready-to-use configuration. The protocol involved a simple, automated process with results available in one hour. During the assay, patient serum is incubated in antigen-coated wells, followed by automated washing steps and the addition of a chemiluminescent conjugate. The resulting luminescence is measured automatically, with the intensity directly proportional to the amount of CAGTA IgG present in the sample. Results are expressed as the following index values: <0.9 negative; 0.9–1.1 equivocal; >1.1 positive.

### 2.3. Wako β-D-Glucan Test

The Wako β-D-glucan assay (Fujifilm Wako Chemicals, Richmond, VA, USA) (Wako BDG) was used to detect β-D-glucan levels in the serum of the patients. This assay is based on a kinetic turbidimetric technique for the detection of fungal cell wall components. Serum samples were collected from patients on the same day as the samples for BCs and stored at −20 °C until analysis. The assay was performed according to the manufacturer’s instructions. The assay was carried out on the Wako analyzer, where patient serum is mixed with reagents and the turbidity change is automatically measured. The intensity of the turbidity change is directly proportional to the amount of β-D-glucan in the sample. The results were interpreted based on kinetic measurements, with a cut-off value determined according to the manufacturer’s guidelines at >7 pg/mL.

### 2.4. CandId Real-Time PCR

The CandId Real-Time PCR assay (OLM diagnostics, Braintree, UK) (CandId PCR) was used to detect *Candida* DNA in the patient’s serum. This assay uses real-time polymerase chain reaction (PCR) technology to detect the following six main causative species associated with candidemia: *C. albicans*, *C. glabrata*, *C. parapsilosis*, *C. tropicalis*, *C. dubliniensis*, and *C. krusei*. Serum samples were collected from patients on the same day as the samples for BCs and stored at −20 °C until analysis. DNA extraction was performed from 0.5 mL of serum using the Nuclisens easyMAG automated system (BioMérieux, Marcy-l’Étoile, France), which is designed for high-throughput sample preparation. The extracted DNA was then used as a template for PCR amplification, following the manufacturer’s instructions. An internal control was included from the DNA extraction step as recommended by the PCR kit insert. The PCR amplification process was carried out using a Biorad CFX96 thermocycler (Bio-Rad Laboratories, Hercules, CA, USA). The serum samples were considered negative if the cycle threshold (ct) was >45.

### 2.5. Statistical Analysis

To evaluate the diagnostic performance of each biomarker, we calculated the sensitivity and specificity. The true positives were defined as patients with *Candida* spp. positive BCs (*n* = 35), while the true negatives were the 20 selected patients with a negative BCs in the same study period. The following cut-off values were used for the respective tests, CAGTA: ≥0.9 (equivocal and positives results), Wako BDG: ≥7 pg/mL, and CandId PCR: ct < 45. Receiver operating characteristic (ROC) curves were generated for CAGTA, Wako BDG, and CandId PCR to assess their diagnostic accuracy and determine the optimal cut-off values. The area under the curve (AUC), along with 95% confidence intervals, was calculated to compare the performance of the biomarkers. We considered a P value of <0.05 to be statistically significant. All statistical calculations were performed using MedCalc^®^ Statistical Software 23.0.2 (MedCalc Software Ltd., Ostend, Belgium; “https://www.medcalc.org” (accessed on 15 January 2025).

## 3. Results

Between August 2023 and January 2025, 35 cases of candidemia were confirmed through positive blood cultures. Table 1 summarizes the demographic data, clinical characteristics of patients with positive and negative blood cultures included in this study, and the distribution of *Candida* species identified in positive blood cultures.

Table 2 summarizes the diagnostic performance of the biomarkers used in this study, including sensitivity, specificity, and the area under the curve (AUC). Wako BDG achieved the best results, followed by CandId PCR and the combination of Wako BDG and CandId PCR. We observed a higher sensitivity of CAGTA in patients with probable deep-seated candidiasis (9 positive cases out of 12, sensitivity 75%) compared to patients without deep-seated candidiasis (7 positive cases out of 23, sensitivity 30%).

**Figure 1:**
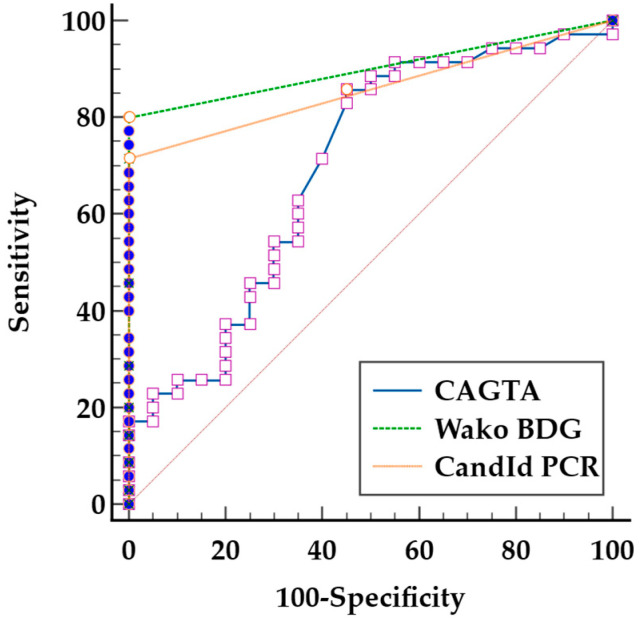
Receiver operating characteristic curves of biomarkers for the diagnosis of IC. Invasive Candidiasis (CAGTA) IgG VirClia Monotest^®^, Wako β-D-Glucan Test^®^, and CandId Real-Time PCR^®^. The gray diagonal line represents the performance of a random classifier with no discriminative power.

We calculated the receiver operating characteristic (ROC) curves for CAGTA, Wako BDG, and CandId PCR to assess their diagnostic accuracy and determine the optimal cutoff values (Figure 1). Pairwise comparisons of the ROC curves revealed that CandId PCR and Wako BDG exhibit similar diagnostic performance (difference between areas [DBA] = 0.04; *p* = 0.4). However, both CandId PCR and Wako BDG demonstrate superior performance compared to CAGTA, although the differences are statistically significant only between Wako BDG and CAGTA (CandId PCR vs. CAGTA: DBA = 0.16; *p* = 0.06 and Wako BDG vs. VirClia CAGTA: DBA = 0.2; *p* = 0.02). The optimal cut-offs proposed by ROC curve calculation for VirClia CAGTA, Wako BDG, and CandId PCR were index > 0.36, > 4.1 pg/mL, and ct < 40, respectively. 

Pairwise comparisons of AUCs revealed no statistically significant differences in biomarker performance between C. albicans (*n* = 18) and non-albicans (*n* = 17) candidemia. The differences in AUCs were 0.01 for CAGTA (*p* = 0.9), 0.01 for Wako BDG (*p* = 0.8), and 0.13 for CandId RT-PCR (*p* = 0.08). The difference between the AUCs for *Candida albicans* and *Candida glabrata* candidemia is statistically significant (difference of 0.2; *p* = 0.02). Table 3 summarizes the percentage of positive results for each biomarker according to the *Candida* species involved in candidemia.

CandId PCR detected both *C. albicans* and *C. dubliniensis* in a patient with candidemia attributed to *C. albicans*, though *C. dubliniensis* was not recovered by blood culture. Additionally, CandId PCR identified *C. albicans* in the patient with candidemia caused by *C. dubliniensis*.

## 4. Discussion

In this study, we retrospectively evaluated the diagnostic performance of the following three serum biomarkers for detecting candidemia in at-risk patients, VirClia CAGTA, Wako BDG, and CandId PCR. CAGTA VirClia assay was originally designed to detect antibodies against *C. albicans* hyphal forms. While cross-reactivity with other *Candida* spp. has been documented, the assay has also been evaluated for detecting antibodies against non-*albicans Candida* species. Nevertheless, its diagnostic performance for these species remains less well established. Additionally, prior exposure to *Candida* spp. could lead to pre-existing IgG levels, potentially affecting the test’s sensitivity and specificity [5]. Furthermore, the presence of CAGTA in candidemia patients has been linked to deep-seated candidiasis (DSC) in some studies. The CAGTA test detects antibodies against a hyphal surface antigen expressed during tissue invasion and biofilm formation, making it potentially helpful in distinguishing between candidemia with and without deep-seated candidiasis. Approximately 50% of candidemia cases result in DSC, which is associated with a worse prognosis due to the challenges of eradicating deep-tissue infections, increased risk of severe complications, and the need for more aggressive or prolonged antifungal therapy. Martinez-Jimenez et al. found that CAGTA was present in 68% of candidemia cases with DSC, compared to only 4.7% in non-DSC cases [11,12,13]. Better performance has been noted in ICU patients, with a meta-analysis showing 66% and 76% pooled sensitivity and specificity, respectively [3,4,14]. Differentiating candidemia with and without DSC is clinically challenging due to nonspecific symptoms, and obtaining deep-tissue samples for confirmation is often not feasible. In our study, the overall sensitivity of the CAGTA test was low at 46%, consistent with previous reports [4,12]. DSC was diagnosed in 34% of the patients, and the sensitivity of CAGTA reached 75%. Given these limitations, CAGTA should not be used as a standalone test but in combination with other biomarkers. Several studies have shown improved sensitivity (90–97%) when CAGTA is combined with β-D-glucan [15,16]. In our study, combining VirClia CAGTA with either Wako BDG or CandId PCR increased sensitivity (86–83%, respectively) for diagnosing candidemia, and when all three biomarkers were used together, sensitivity reached 94%.

β-D-glucan (BDG) is a broad-spectrum fungal biomarker that has demonstrated high effectiveness in diagnosing invasive fungal infections (IFIs), particularly candidemia and pneumocystosis, due to its strong negative predictive value (NPV). This makes BDG a valuable tool for ruling out fungal infections in patients with suspected IFIs [17,18,19]. In our study, Wako BDG showed a sensitivity of 74%, which increased to 86-94% when combined with other biomarkers, as previously discussed. The best performance was achieved by combining Wako BDG and CandId PCR, with a sensitivity of 91% and a specificity of 100%. Evaluating the specificity of BDG is challenging due to its pan-fungal nature. The combination of multiple biomarkers aims to enhance diagnostic accuracy, but it is essential to recognize that when BDG is included, the detection is not strictly limited to candidemia. This must be carefully interpreted, particularly when analyzing combined test results in the context of invasive fungal infections. However, BDG was not detected in any of the 20 patients with negative blood cultures in this study. The literature reports variable BDG performance in diagnosing invasive candidiasis, with sensitivities ranging from 42.5% to 91% and specificities between 97% and 98%, depending on the cut-off values used [20,21,22]. Reduced sensitivity (73%) and specificity (45%) have also been observed in ICU patients with invasive candidiasis [23]. 

PCR-based assays have gained increasing importance in the diagnosis of invasive candidiasis due to their ability to rapidly and accurately detect fungal DNA from common *Candida* species in clinical samples. Among these, the T2 *Candida* test (T2 Biosystems, Wilmington, MA, USA) is an advanced diagnostic tool that detects *Candida* species directly from whole blood without requiring blood cultures, offering rapid results with high sensitivity and specificity. This makes it particularly valuable for the early detection of candidemia. However, despite its strong performance, the T2 Candida test has limitations, including high cost and limited availability in many healthcare settings [12,24,25]. In comparison, other multiplex PCR assays, such as the OLM CandId PCR test, provide a more cost-effective alternative, though they have not been as extensively evaluated as T2 *Candida*. Price et al. conducted a real-time PCR evaluation of the OLM CandId kit for diagnosing invasive candidiasis, reporting a sensitivity of 80% and a specificity of 93%, consistent with our own findings [26]. In our study, the sensitivity of CandId PCR was slightly lower than reported in the literature, though it maintained excellent specificity and performed slightly below that of β-D-glucan. Sensitivity was lower in candidemia caused by *Candida* non-*albicans* species compared to *C. albicans* candidemia (59% versus 83), although this difference was not statistically significant. This sensitivity was significantly lower for *Candida glabrata* candidemia (44%). Considering that *C. glabrata* candidemia is the second most common in our setting and is often associated with antifungal resistance, efforts should be made to improve its detection for routine clinical use [27]. Although the OLM CandId PCR offers lower cost and wider accessibility, further studies are needed to define its role in the diagnostic landscape better.

The co-detection of *C. albicans* and *C. dubliniensis* by the CandId PCR underscores a key advantage of this method due to its ability to detect co-infections which are more challenging to identify using traditional methods. In this case, a false-positive detection of *C. dubliniensis* is a more likely explanation than a failure of the culture to isolate it. Given the close phylogenetic relationship between *C. albicans* and *C. dubliniensis*, the dual detection likely reflects this similarity [28]. Furthermore, the observation that the only *C. dubliniensis* candidemia in our study tested positive by PCR for *C. albicans* reinforces this hypothesis. The CandId PCR assay is the only technique in our study designed to directly detect *Candida* non-*albicans* species. Nevertheless, the number of detectable species in PCR-based assays, such as T2 Candida or CandId PCR, is limited. This raises the risk of false negatives in cases of candidemia caused by species not included in the assay. These undetected species could include *Candida* or other yeast species with antifungal resistance, potentially impacting patient management and survival. The inability to identify resistant pathogens may delay appropriate therapy, thereby worsening outcomes. This is exemplified in our patients with *C. guillermondii* candidemia, and with co-infection involving *Candida glabrata* and *Magnusiomyces capitatus*.

Antifungal exposure can reduce the performance of biomarkers [12]. In our study, only three patients were on antifungal prophylaxis (fluconazole). In these cases, CandId PCR and CAGTA yielded negative results, while BDG was positive. This observation underscores the potential limitations of certain biomarkers in patients receiving antifungal treatment and highlights the importance of considering antifungal exposure when interpreting diagnostic results.

Our study has several limitations that should be considered. First, we acknowledge that our sample size is modest (35 candidemia cases over more than one year), which may limit the generalizability of our findings. Additionally, the freezing and thawing of serum samples may have affected biomarker accuracy, potentially introducing variability in the results. Another limitation is the retrospective classification of patients with deep-seated candidiasis based on clinical records, which presents inherent challenges. Diagnosing deep-seated infections is notoriously difficult in clinical practice, and misclassification cannot be ruled out. Despite these challenges, we included these patients to address this complex subgroup; however, a prospective study focusing specifically on deep-seated candidiasis would provide more robust data to evaluate biomarkers like CAGTA. Lastly, we were unable to assess biomarker performance in cases of invasive candidiasis not detected by blood cultures, leaving a gap in understanding their diagnostic utility in such scenarios.

## 5. Conclusions

The diagnosis of candidemia via blood cultures faces two major limitations: prolonged incubation times and low sensitivity. Consequently, combining blood cultures with serological biomarkers could enable faster and more reliable detection, which is crucial for improving patient outcomes. Our study highlights the limited effectiveness of CAGTA and the moderate performance of both the Wako BDG and CandId PCR assays. Notably, the best diagnostic performance was achieved when Wako BDG and CandId PCR assays were combined, suggesting that a multi-marker approach can enhance both sensitivity and accuracy, albeit at a higher cost. However, despite this improvement, these biomarkers remain insufficient as standalone diagnostic tools in routine practice. Larger prospective studies are needed to validate these findings and further assess the clinical utility of these biomarkers across diverse patient populations and *Candida* species.

## Figures and Tables

**Table 1:** Demographic and clinical characteristics of patients with positive and negative blood cultures.

	Positive BD ^1^ (n = 35)	Negative BD ^1^ (n = 20)
**Demographics**		
Age (median years, range) Gender (male/female)	69 (21–90)	68 (23–90)
	21/14	14/6
**Hospitalization Units ^2^**		
ICU ^3^ (%) Onco-Hematology (%) Digestive Surgery (%) Internal Medicine (%) Urology (%) Geriatrics (%) Others (%)	14 (40%)	1 (5%)
	5 (14%)	4 (20%)
	4 (11%)	1 (5%)
	4 (11%)	9 (45%)
	3 (9%)	-
	3 (9%)	4 (20%)
	2 (6%)	1 (5%)
**Hospital admission reasons**		
Surgery (%) Severe bacterial infection (%) Febrile neutropenia (%) Gastrointestinal obstruction (%) Neoplastic recurrence (%) Viral infection (%) Others (%)	12 (34%)	5 (25%)
	8 (23%)	5 (25%)
	4 (11%)	1 (5%)
	4 (11%)	-
	3 (9%)	1 (5%)
	2 (6%)	4 (20%)
	2 (6%)	4 (20%)
**Antifungal exposure ^4^**	3 (9%)	-
**One-month mortality**	15 (43%)	3 (15%)
**Probable deep-seated candidiasis**	12 (34%)	-
***Candida* spp distribution ^5^**		
*Candida albicans* *Candida glabrata* ^6^ *Candida tropicalis* *Candida krusei* *Candida guillermondii* *Candida dubliniensis*	18 (51%)	-
	9 (26%)	-
	5 (14%)	-
	1 (3%)	-
	1 (3%)	-
	1 (3%)	-

^1^ Blood culture. ^2^ Hospitalization unit at the time of blood culture. ^3^ Intensive Care Unit. ^4^ Antifungal exposure at the moment of blood culture. ^5^ *Candida* spp. distribution of positive blood culture. ^6^ Concurrent isolation of *Candida glabrata* and *Magnusiomyces capitatus* from a patient.

**Table 2:** Sensitivities and specificities.

Biomarker	Sens ^1^ (%) (95% CI ^4^)	Spec ^2^ (%) (95% CI)	AUC ^3^ (95% CI)
**CAGTA ^5^**	46 (29–63)	75 (51–91)	0.6 (0.4–0.7)
**BDG ^6^**	74 (57–87)	100 (83–100)	0.9 (0.7–0.9)
**PCR ^7^**	71 (54–85)	100 (83–100)	0.9 (0.7–0.9)
**CAGTA and/or BDG**	86 (70–95)	75 (51–91)	0.8 (0.7–0.9)
**CAGTA and/or** **PCR**	83 (66–93)	75 (51–91)	0.8 (0.7–0.9)
**BDG and/or** **PCR**	91 (77–98)	100 (83–100)	0.9 (0.8–1)
**CAGTA and/or BDG and/or PCR**	94 (81–99)	75 (51–91)	0.8 (0.7–0.9)

^1^ Sensitivity. ^2^ Specificity. ^3^ Area Under the Curve. ^4^ Confidence interval invasive. ^5^ Candidiasis (CAGTA) IgG VirClia Monotest (cut-off value ≥ 0.9). ^6^ Wako β-D-Glucan Test (cut-off value ≥ 7 pg/mL). ^7^ CandID Real-Time PCR (cut-off value ct ≥ 45).

**Table 3:** Percentage of positive results for each biomarker according to *Candida* spp.

*Candida* spp. Candidemia	CAGTA ^1^	Wako BDG ^2^	CandId PCR ^3^
*Candida albicans* (*n* = 18)	10 (55%)	14 (78%)	15 (83%)
*Candida glabrata* (*n* = 9)	4 (44%)	8 (89%)	4 (44%)
*Candida tropicalis* (*n* = 5)	2 (40%)	3 (60%)	4 (80%)
*Candida krusei* (*n* = 1)	0 (0%)	0 (0%)	1 (100%)
*Candida guillermondii* (*n* = 1)	0 (0%)	0 (0%)	0 (0%)
*Candida dubliniensis* (*n* = 1)	0 (0%)	1 (100%)	1 (100%) ^4^

^1^ Candidiasis (CAGTA) IgG VirClia Monotest). ^2^ Wako β-D-Glucan Test. ^3^ CandID Real-Time PCR. ^4^ CandId PCR positive for *Candida albicans.*

## Data Availability

The original contributions presented in this study are included in the article. Further inquiries can be directed to the corresponding authors.
